# Characterization of Sex Differences in Ocular Herpes Simplex Virus 1 Infection and Herpes Stromal Keratitis Pathogenesis of Wild-Type and Herpesvirus Entry Mediator Knockout Mice

**DOI:** 10.1128/mSphere.00073-19

**Published:** 2019-03-27

**Authors:** Rachel E. Riccio, Seo J. Park, Richard Longnecker, Sarah J. Kopp

**Affiliations:** a Department of Microbiology and Immunology, Northwestern University Feinberg School of Medicine, Chicago, Illinois, USA; University of Arizona

**Keywords:** herpes simplex virus, herpes stromal keratitis, ocular, sex differences

## Abstract

Sex differences related to immune response and inflammation play a role in the susceptibility and pathogenesis of a variety of viral infections and disease (S. L. Klein, Bioessays 34:1050–1059, 2012, https://doi.org/10.1002/bies.201200099). Herpes simplex virus 1 (HSV-1) causes chronic inflammatory disease in the cornea, an immune-privileged tissue, resulting in irreversible damage and blindness in affected individuals (A. Rowe, A. St Leger, S. Jeon, D. K. Dhaliwal, et al., Prog Retin Eye Res 32:88–101, 2013, https://doi.org/10.1016/j.preteyeres.2012.08.002). Our research focuses on the role of herpesvirus entry mediator (HVEM) as an immune regulator during ocular HSV-1 infection. Mice lacking HVEM (HVEM knockout [KO] mice) exhibit lower levels of immune cell infiltrates and less severe ocular disease in the cornea than wild-type (WT) mice. As sex differences contribute to pathogenesis in many inflammatory diseases, we tested whether sex acts as a biological variable in the immune response to HSV-1 infection and herpes stromal keratitis (HSK) pathogenesis. Adult male and female WT and HVEM KO mice were inoculated with HSV-1 via corneal scarification and monitored daily for disease course. Viral titers were determined, and immune cell infiltrates were collected and analyzed. Our results indicated no significant differences in viral titers in tear film or affected tissues, in immune cell infiltration, or in clinical symptoms between males and females of either genotype. These results suggest that sex is not a significant biological variable in this experimental model and that male and female mice of the C57BL/6 background can be used similarly in studies of ocular HSV-1 pathogenesis.

**IMPORTANCE** Sex hormones have come to be considered an important factor for the development of certain diseases only recently and as such should continue to be considered a biological variable. Ocular HSV-1, and the resulting HSK, is the leading cause of infectious blindness worldwide. We compared levels of ocular HSV-1 infection and pathogenesis in the two sexes and found no significance differences between male and female WT mice or HVEM KO mice.

## Abstract (precis)

Sex hormones have come to be considered an important factor for the development of certain diseases only recently and as such should continue to be considered a biological variable. Ocular HSV-1, and the resulting HSK, is the leading cause of infectious blindness worldwide. We compared levels of ocular HSV-1 infection and pathogenesis in the two sexes and found no significance differences between male and female WT mice or HVEM KO mice.

## INTRODUCTION

Sex differences play a role in the pathogenesis of a variety of viral infections, including influenza A virus, Epstein-Barr virus, hepatitis B and C virus, and herpes simplex virus (HSV) infections (1, 2). In studies of such infections, differences in the susceptibility and severity of the viral infections between males and females have been attributed to sex hormones which have roles in promoting immune cell function and cytokine synthesis (1–4). Notably, an epidemiological human study of HSV type 2 (HSV-2) infections showed that women have a higher acquisition rate, a higher incidence of symptoms, and a higher prevalence of infection than men in genital infections (2, 5, 6). Whereas women have a greater risk of HSV-2 due to biologic susceptibility, men have a greater recurrence rate than women (5, 7). However, the rates and recurrences of HSV type 1 (HSV-1) infections are similar in men and women in human genital infection (5). In a murine model of resistance to HSV-1, researchers found a sex-based difference in resistance in mice with the herpes resistance locus (Hrl), which is a part of the tumor necrosis factor (TNF) superfamily, when the mice were challenged via ocular scarification with HSV-1; 52% of the males were resistant to HSV-1 induced mortality, compared to 68% of the female mice (3). Although a clear sex bias has been shown in HSV-2 infections, the differences in HSV-1 pathogenesis between males and females remain unclear.

Our laboratory studies herpesvirus stromal keratitis (HSK), which is an ocular disease that is the leading cause of infectious blindness worldwide (8, 9). It is typically caused by HSV-1 (10, 11). In our current study, we investigated if sex plays a role in this disease using a murine model. HSV-1 is a large double-stranded DNA virus that enters and replicates in host cell mucosal membranes (12, 13). The virus then travels from the mucosal surfaces to sensory neurons, establishes latency, and persists in a latent state for the lifetime of the host. Reactivation can occur at any time with replication of virus traveling back to the mucosal surfaces. During ocular infection, reactivation of the virus from latency in the trigeminal ganglion (TG) to the corneal epithelium causes an inflammatory response characterized by corneal opacity and neovascularization which can ultimately lead to blindness due to these periodic episodes of virus reactivation (13–15).

Our previous studies have shown that herpesvirus entry mediator (HVEM), a member of the TNF superfamily, has immunomodulatory functions and promotes ocular HSV-1 pathogenesis independently of viral entry receptor functions (16, 17). HVEM is a key immune regulatory protein that is part of the HVEM/LIGHT/BTLA/CD160 cosignaling pathway that regulates T-cell activation and function (7). HVEM has a role in the adaptive immune responses of murine corneal inflammation and nerve damage that occur following ocular challenge of HSV-1, which is critical for the development of HSK (8). Mice lacking HVEM (HVEM knockout [KO] mice) exhibit lower levels of immune cell infiltrates and less severe ocular disease in the cornea than C57BL/6 wild-type (WT) mice (8). Since a clear sex difference in rates of mortality was observed in ocular studies with Hrl, we tested whether sex factors into our model of HSV-1 ocular infection. Moreover, sex hormones have been found to affect the expression of host cell surface proteins that function as receptors for viral entry such as the chemokine receptors for HIV-1, or DAF, a receptor for coxsackievirus. In these cases, estrogen promotes increased expression of receptors on B cells, which correlates with increased susceptibility of cells to infection and subsequent replication (2, 18, 19). In addition, immune responses to pathogen infection can also differ between the sexes. Most pertinent to our studies are those that show changes in cytokine expression which alters the immune response in comparisons of males to females. For example, when peripheral blood mononuclear cells from healthy men and women were stimulated with HSV-1 or Toll-like receptor 9 (TLR9) ligands, interleukin 10 (IL-10) production was found to be statistically significantly related to plasma levels of sex hormones in both groups (12). Men produced higher levels of IL-10, which serves as a negative regulator of the response of both innate and adaptive immune cells under conditions of stimulation with HSV-1 or TLR9 ligands, than women (12). Previously, we used only male mice in our studies for characterizing HSK pathogenesis and found that HVEM modulates the immune response to ocular HSV-1 infection. Since sex has also been shown to play a role in the immune response to viral infection, we investigated whether female mice of the WT and HVEM KO genotype exhibit a response to ocular HSV-1 infection similar to that exhibited by males to determine if sex is a biological variable in HSV-1 infection and HSK pathogenesis. Moreover, little is known in regard to the role of sex in ocular health and disease (2, 10, 12).

Findings of previous studies exploring the role of sex using different murine models of ocular HSV-1 have been contradictory. The experimental models have used other routes of infection, virus and mouse strains, and ages at infection. In murine models of ocular HSV-1 infection, sex has been shown to play a role in only certain mouse backgrounds (14, 20). Female mice of the 129/Sv/Ev background displayed lower clinical periocular disease scores than both males and testosterone-treated females although the viral replication and corneal disease conditions were similar, suggesting that sex hormones enhance the severity of the clinical symptoms of HSK (20). In contrast, male and female mice of the NIH/OLA background displayed similar clinical scores and viral titers from the trigeminal ganglia, indicating that sex does not play a role in pathogenesis of HSK in NIH/OLA background (21). Studies have also found sex differences in response to the HSV-1 glycoprotein D vaccine being more effective in women than in men due to differences in immune cell response, where the gD peptide epitopes produced a higher CD4^+^ T-cell response in females than males (22). Exploring the relationship between sex hormones and viral infection may offer insights into treatment and vaccination and a better understanding of the differences in immune cell responses of males and females that may lead to the development of novel therapies for human autoimmune diseases. Autoimmune diseases are more prevalent in women than in men (1, 2). Eight percent of the population is affected by autoimmune diseases, and over two-thirds of those affected are woman. The stronger innate and adaptive immune response in females contributes to their increased susceptibility to inflammatory disease, and understanding whether this occurs in ocular HSV-1 infection and pathogenesis may suggest treatments that lessen the severity of disease in humans (23).

In our current studies, we investigated sex-specific differences between male and female mice in HSK pathogenesis in the C57BL/6 mouse strain to determine if sex plays a role in the inflammatory disease induced by the HVEM/LIGHT/BTLA/CD160 cosignaling pathway that regulates T-cell activation and function (7). Our results indicate that there was no significant difference between male and female mice in evaluations of HSK disease pathogenesis. Our findings are informative in determining the role of sex in this disease and allowing the use of both male and female mice in our ongoing studies and future experiments using the C57BL/6 mouse strain, which is commonly used for genetic knockout mouse lines.

## RESULTS

### HSV-1 replicates to similar levels in male and female WT and HVEM KO mice after corneal inoculation.

**FIG 1:**
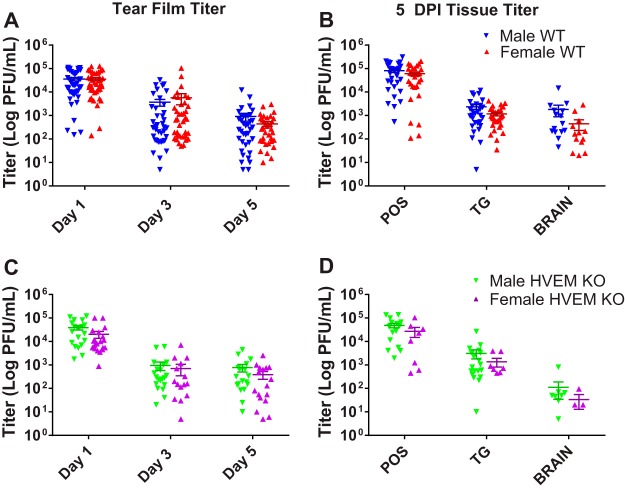
Male and female WT and HVEM KO mice exhibit similar HSV-1 eye swab results and tissue titers after corneal inoculation. Eye swabs were collected at 1, 3, and 5 dpi, and periocular skin (POS), trigeminal ganglia (TG), and brain tissues were collected at 5 dpi. (A) Titers from eye swabs of male and female WT mice (*n* = 5 to 10 mice per group, 3 replicates). (B) Titers from tissues of male and female WT mice at 5 dpi (*n* = 4 to 5 mice per group, 3 replicates). (C and D) Titers from eye swabs of male and female HVEM KO mice (*n* = 5 to 10 mice per group, 3 replicates) (C) and from tissues of male and female HVEM KO mice (*n* = 3 to 5 mice per group, 2 replicates) (D). No difference was observed between the two sexes in each genotype. Titers calculated as means ± standard errors of the means (SEM) were analyzed using two-tailed *t* tests with Holm-Sidak's correction for multiple comparisons.

In a murine model of infection, primary infection of the corneal epithelium resulted in detectable replication of HSV-1 for up to 6 days postinfection (dpi) in studies monitoring ocular tear film (11, 15). To determine whether sex impacts the replication of HSV-1, age-matched male and female adult WT and HVEM KO mice were inoculated with HSV-1 strain 17 via corneal scarification and viral titers of eye swabs of tear film were collected at days 1, 3, and 5 postinfection. The titers showed no significant difference between male and female mice of either the WT or HVEM KO genotype at all 3 time points (Fig. 1A and C). Female WT mice had slightly higher titers than WT males on dpi 3 and slightly lower titers than males on dpi 5 (Fig. 1A). Female HVEM KO mice had slightly lower eye swab titers than male HVEM KO mice on all 3 days (Fig. 1C). In all cases, these differences were not statistically significant.

Following ocular infection, active replicating virus can also be quantified in the brain and surrounding tissues during the acute phase of infection since it disseminates by retrograde transport to the neuronal cell bodies in the trigeminal ganglia (8, 11). To monitor dissemination, we collected periocular skin (POS), trigeminal ganglia, and brain tissues at 5 days postinfection to study this phase of the HSV-1 infection cycle (Fig. 1B and D). Although there was no significant difference between the tissue titers in male and female mice within either genotype, lower titers were observed in tissues collected from female mice overall, but the differences from the results seen with the male mice were not statistically significant. From these data, we determined that sex has no significant effect on HSV-1 viral replication in the cornea, periocular skin, trigeminal ganglia, or brain of WT and HVEM KO mice.

### Male and female WT and HVEM KO mice have similar cellular corneal immune infiltrates during the acute and chronic inflammatory phases of infection.

We next investigated the corneal cellular immune response in WT and HVEM KO mice to determine if there were any sex differences in the prominent infiltration of inflammatory cells that occurs in stromal tissues following HSV-1 infection of the cornea. Previously, we found that the cytokine responses in HVEM KO male mice were different from those in WT male mice (7, 8). Male and female WT mice exhibited similar levels of HVEM expression during HSV-1 infection after corneal inoculation on dpi 5 and 14 and on immune cells, and male and female HVEM KO mice lacked HVEM expression, as expected (data not shown).The early response to infection while the virus is still actively replicating consists of polymorphonuclear leukocytes (PMN), mainly neutrophils, and is known as the acute inflammatory phase of infection. PMN infiltration peaks at around 2 dpi, declining at 5 dpi and rising again during the chronic inflammatory phase of infection starting around 7 dpi, after the virus has been cleared. Macrophages, dendritic cells (DCs), and natural killer cells are also detected during this time, and T cells enter the cornea during the chronic phase at 8 to 9 dpi (11). HSK is caused by this immunopathogenic response to infection, and differences in infiltrating immune cell populations affect the overall severity of symptoms in the cornea (11, 15). An innate difference in cell-mediated immune responses exists between males and females, including the responses to viral infection. The degrees of reliance on subsets of CD4 helper T cells in overcoming infection differ between males and females, where the levels of Th1 and interferon gamma (IFN-γ) responses in females have been reported to be higher than those in males (2, 24, 25). Androgens have been found to enhance viral and immune factors, where estrogens modulate both innate and adaptive immunity to be protective (26). Further investigation into whether higher levels of estrogens or progesterone, such as during pregnancy, might affect ocular infection should be considered.

**FIG 2:**
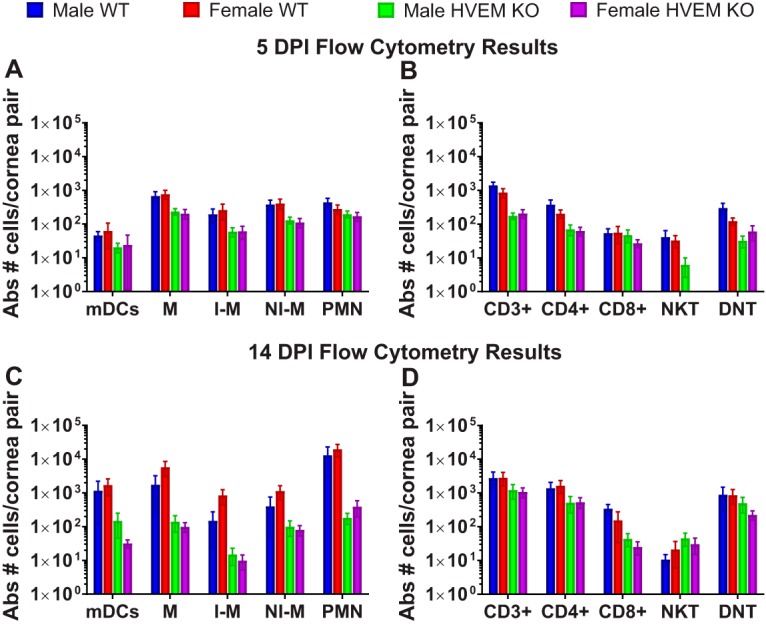
Immune cells infiltrate male and female WT and HVEM KO corneas at similar levels during the acute (5-dpi) and chronic (14-dpi) phases of HSV-1 corneal infection. Data represent results of flow cytometry analysis of immune cell infiltrates in WT and HVEM KO corneas collected at 5 dpi (*n* = 8 to 10, two replicates) (A and B) and 14 dpi (C and D). (A and C) Absolute (Abs) number of myeloid infiltrates of dendritic cells (mDCs), macrophages (M), inflammatory macrophages (I-M), noninflammatory macrophages (NI-M), and polymorphonuclear leukocytes (PMN) at 5 dpi (A) and at 14 dpi (C). (B and D) Absolute number of lymphoid infiltrates of CD4^+^ T cells, CD8^+^ T cells, NK T cells, or DN T cells per cornea pair at 5 dpi (B) and at 14 dpi (D) (*n* = 8 to 20 mice, 2 or 3 replicates). No NKT cells were detected in female HVEM KO corneal samples at 5 dpi. There was no significant difference between males and females of either genotype. The differences between the WT and HVEM KO data are consistent with our previous studies. Infiltrating cell percentages (calculated as means ± SEM) were analyzed using two-tailed *t* tests with Holm-Sidak's correction for multiple comparisons.

Cornea pairs were collected from infected male and female WT and HVEM KO mice during the acute phase of infection at 5 dpi (Fig. 2A and B) and then at the chronic inflammatory phase of infection at 14 dpi (Fig. 2C and D) to determine if the immune cell populations infiltrating the corneas of male and female adult mice differed during either phase of infection. Cell populations were measured via flow cytometry as described in Materials and Methods by first gating on populations of CD45^+^. The counts of infiltrating cells from CD45^+^/CD11b^+^ myeloid lineages during the acute inflammatory phase of infection were determined (Fig. 2A). These populations included Ly6G^−^/CD11c^+^ myeloid dendritic cells (mDCs), Ly6G^−^/CD11c^−^ monocytes and macrophages (M), Ly6G^−^/CD11c^−^/Ly6C^+^ inflammatory macrophages (I-M), Ly6G^−^/CD11c^−^/Ly6C^−^ noninflammatory macrophages (I-M), and Ly6G^+^/CD11c^+^ polymorphonuclear leukocytes (PMN). The counts of infiltrating cells from CD45^+^/CD3^+^ lymphoid lineages during the acute phase were also determined (Fig. 2B). These populations include CD4^+^ CD8^−^ CD4 T cells (CD4^+^), CD4^−^ CD8^+^ CD8 T cells (CD8^+^), NK1.1^+^ natural killer T cells (NKT), and CD4^−^ CD8^−^ NK1.1^−^ double-negative T cells (DNT). The myeloid and lymphoid cell populations during chronic inflammatory phase were also measured (Fig. 2C and D). We analyzed these specific populations, including the CD4^+^ T cells and PMN neutrophils, because they contribute to most of the damage to the eye during HSK (11). We found, as expected, that the WT male mice had significantly higher numbers of PMN cells and CD4^+^ T cells during the chronic phase of infection than the HVEM KO male mice (8). This difference between genotypes was also observed in the female mice. Overall, the male and female mice exhibited similar levels of immune cell infiltration in each genotype. The data suggest that sex does not affect immune cell infiltrates in the cornea during HSV-1 ocular infection.

### Clinical symptoms of corneal HSV-1 pathogenesis are similar in male and female WT and HVEM KO mice.

**FIG 3:**
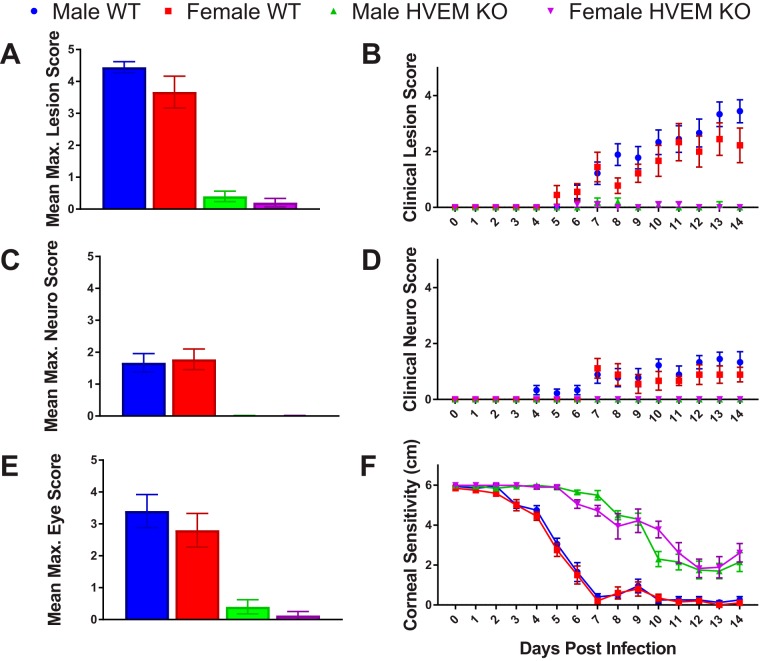
Male and female mice exhibited similar clinical symptoms during the chronic inflammatory phase of HSV-1 corneal infection. Clinical symptoms were scored for each mouse and recorded each day from inoculation to the chronic inflammatory phase (days 0 to 14). Mice were scored on the severity and presence of lesions (A and B), neurological (Neuro.) disease (C and D), and eye disease (E), as well as on corneal sensitivity (F) and weight loss (not shown). (A) Maximum (Max.) lesion score within 14 dpi. (C) Maximum neurological disease score within 14 dpi. (E) Maximum eye disease score within 14 dpi. (B) Lesion score from 0 to 14 dpi. (D) Neurological disease score from 0 to 14 dpi. (F) Corneal sensitivity from 0 to 14 dpi. *n* = 5 mice per group, 2 replicates. No difference was observed between the sexes. Scores and sensitivity levels (calculated as means ± SEM) were analyzed using two-tailed *t* tests with Holm-Sidak's correction for multiple comparisons.

Mice infected with HSV-1 exhibit not only ocular disease but also neurological symptoms, including seizures, loss of balance, and weight loss. To determine whether sex plays a role in clinical symptoms of HSK and pathogenesis of corneal infection, male and female WT and HVEM KO mice were scored for clinical symptoms daily from 0 to 14 dpi (Fig. 3). As indicated in Table 1, scores were based on appearance and severity of lesions, neurological symptoms, and clinical eye disease on a scale of 0 to 5, with a score of 0 representing the absence of symptoms and a score of 5 representing the most severe symptoms. The mean maximum lesion scores were 4.4 for male WT mice, 3.7 for female WT mice, 0.4 for male HVEM KO mice, and 0.2 for female HVEM KO mice (Fig. 3A). The levels of severity of lesions were similar between males and females within each genotype. The occurrence of lesions and the mean score on each day were also determined (Fig. 3B). Male and female HVEM KO mice experienced similar rates of progression of lesions, and the female WT mice had slightly lower scores than the male WT mice. The neurological scores were recorded for the male and female mice, and the mean maximum score was calculated. The mean maximum neurological scores were 1.7 for male WT mice and 1.8 for female WT mice; HVEM KO mice did not display any neurological symptoms, as we had previously seen (Fig. 3C). The levels of severity of neurological symptoms were similar between the males and females within each genotype. The onset of neurological symptoms and mean score on each day were determined (Fig. 3D). The male and female mice experienced similar levels of neurological symptoms throughout HSV-1 pathogenesis. Additionally, eye disease scores were determined for male and female mice. The mean maximum eye disease scores were 3.4 for male WT mice, 2.8 for female WT mice, 0.4 for male HVEM KO mice, and 0.13 for female HVEM KO mice (Fig. 3E). The levels of severity of eye disease symptoms were also similar between the males and females within each genotype. Overall, the female mice exhibited attenuated clinical symptoms compared to the male mice in both genotypes, but the differences were not statistically significant. Our results obtained with the female and male mice were identical to those seen in our previous studies with male mice, showing that HVEM contributes to increased severity of disease in WT mice compared to HVEM KO mice (16).

**TABLE 1:** Clinical symptom scoring scale for HSK in mice

Score	Description of score by type		
	Lesion	Neurological	Eye
0	Lesion free	Symptom free	Symptom free
1	Small area of broken skin of <0.5 cm	Ruffled fur, hunched posture, normal movement	Pus around edge(s)
2	Area of broken skin of 0.5–1 cm	Hunched posture, slow movement	Pus and squint
3	Broken skin, bleeding, scabbing, or pustules	Hunched posture, slow movement, labored breathing	Closed
4	Broken skin of >1 cm with multiple pustules or scabbing	Hunched, labored breathing, little to no movement	Closed, scab formation
5	Severe scabbing or bleeding with pustules	Moribund or dead	Severe scabbing

A major symptom of HSK includes corneal scarring, which leads to loss of corneal sensitivity and vision. Esthesiometry, which measures corneal sensitivity determined on the basis of the shortest filament length required to elicit a blink response, was used to monitor corneal sensitivity from 0 to 14 dpi, with a filament 6 cm in length representing no loss of sensitivity and a filament 0.5 cm or less in length representing total loss of sensitivity (Fig. 3F). Male and female mice had similar levels of corneal sensitivity throughout corneal infection. Our previous data showing that HVEM contributes to loss of corneal sensitivity were determined by comparing HVEM KO to WT adult male mice, where WT mice lost sensitivity and HVEM KO mice retained sensitivity (8). The same result was observed in female mice. We conclude from this that sex does not play a significant role in the clinical disease course during HSV-1 ocular infection of mice.

## DISCUSSION

Susceptibility to viral infections is often reduced in females compared to males due to a greater humoral and immune response to vaccination and infection (2). Historically, only one sex was used in studies performed with murine models of HSV-1 infection, and male-predominant populations have been used in human studies and trials (2, 10). Studies have shown sex dependence of HSV infection of the central nervous system, where females were more susceptible than males (27, 28). Female mice have also been shown to have higher HSV titers from brain tissue than males when inoculations were performed via intraperitoneal injection, resulting in greater mortality and severity of disease (1, 28). Data representing estrogen treatments, castration, or hormones do not fully explain these sex differences (1, 20). In human studies, the high prevalence of HSV-1 in the trigeminal ganglion (TG) was shown not to be related to sex, with 88% of women and 90% men having HSV-1-positive TG (29), and the incidence rates of HSK were found to be similar in men and women, at a ratio of 1.1 to 1 (30). These studies revealed the importance of addressing and understanding differences in susceptibility to HSV-1 infection and ocular disease between the sexes since such understanding may result in more-effective therapies and vaccines.

Our results indicate that the viral titers in tear film and tissues from adult HVEM KO and WT mice tissue were not affected by sex (Fig. 1). The data revealed a trend showing female mice to have slightly lower tissue titers than male mice of both the WT and HVEM KO genotypes. These findings are similar to those of an ocular model using the 129 mouse strain background, in which the viral titer in the TG was slightly lower in females but not statistically significantly so (20). In contrast, HSV-2 infection has shown a sex bias in human studies in which women exhibited a higher prevalence of infections than males in all age groups, and seronegative women acquired virus faster and had a higher incidence of symptomatic infections than males (2, 6, 31). Our previous research showed that HVEM is not required for disease during HSV-2 infection of mice but that HVEM is required for full HSV-1 pathogenesis in the eye following infection (8). Our data instead suggest that, being influenced by sex hormones as in the case of HSV-2 infection, the immune response in HSV-1 pathogenesis is modulated by HVEM. This may suggest why sex differences have been reported in HSV-2 but were not found in our research of ocular HSV-1 pathogenesis. Another study found some effects of the interferon gamma (IFN-γ) receptor to be sex specific in ocular HSV-1 infection. In IFN-γ receptor knockout mice, females displayed less-severe POS disease scores than males, and testosterone-induced females produced male-like POS symptoms (20). However, the effect of sex hormones on host receptors such as HVEM has yet to be fully understood, and further investigation into what is contributing to the reduced infiltrates and less-severe disease in mice lacking HVEM is needed.

The immune privilege of the cornea, which is the site of infection, may account for the fact that sex hormones do not influence the immune response in HSK. Previous studies have shown the development of corneal eye disease to be strongly correlated with an inflammatory response characterized by CD4^+^ Th1 cytokine-producing T cells (20, 32). The results shown in Fig. 2 representing analysis of CD4^+^ cells showed that males had a higher number of CD4^+^ cells in response to infection during the acute stage whereas females had a slightly higher CD4^+^ response during chronic infection. In the chronic inflammatory stage of infection, which contributes to the severity of HSK, no significant differences were observed in the levels of immune cell infiltrates (Fig. 2C, D). HVEM is known to influence HSV pathogenesis in the ocular murine model during most stages of infection, including entry and acute viral replication, innate responses, chronic inflammation, and viral latency (8, 33–36). Our previous findings showed that HVEM contributes significantly to the increases in the levels of immune cell infiltrates that occur during ocular HSV-1 infection (17). Since no significant differences were observed between males and females within each genotype and since the differences between the genotypes were consistent within the sexes, we conclude sex that does not play a role in the innate or adaptive response to ocular HSV-1 infection.

In addition, the difference in clinical symptoms between WT and HVEM KO mice that we observed in our previous studies confirmed that HVEM modulates the immune response rather than being altered by sex (17). Our results also show no significant differences in clinical symptoms between males and females of either genotype in our experimental model. Female mice trended toward slightly lower clinical scores than male mice in both the WT and HVEM KO genotypes (Fig. 3). A previous study using the HSV-1 KOS strain found that males displayed less-severe clinical symptoms (disease score mean of 1.5) than females (score mean of 2.0) on average (14). This was also observed in another study in which clinical eye disease scores were slightly lower in females but not statistically significantly so (20). Clinical examination using a slit-lamp microscope may provide better diagnosis and scoring of HSK as this is the primary tool for patient diagnosis (37, 38). However, there were no sex differences in the corneal eye disease scores in our results and in the results reported from other studies of ocular HSV-1 infection and clinical HSK pathogenesis (14, 20).

Our results suggest that sex is not a significant biological variable in ocular HSK in the C57BL/6 mouse background and does not contribute to the observed reduction of the levels of immune cell infiltration and severity of clinical symptoms in HVEM KO mice. Overall, we determined that there was no significant difference in HSV-1 ocular infection in male and female mice. In compliance with the NIH standards of considering sex a biological variable, we report that both male and female mice can be used simultaneously in studies using C57BL/6 mice and HVEM KO mice on the C57BL/6 background.

## MATERIALS AND METHODS

### Ethics statement.

All experiments utilizing mice were performed in strict adherence to the recommendations in the Guide for the Care and Use of Laboratory Animals of the National Institutes of Health. The Institutional Animal Care and Use Committee of Northwestern University approved the protocol (Protocol no. IS00001532). Procedures were performed with mice under anesthesia using a ketamine/xylazine cocktail or under isoflurane anesthesia. Minimization of suffering was prioritized.

### Cells and viruses.

African green monkey kidney cells (Vero) were used for propagation of virus and all viral plaque assays. HSV-1 strain 17 was obtained from David Leib (Dartmouth Medical School, Hanover, NH).

### Viral plaque assay.

Viral plaque assay was performed using Vero cells to determine viral titers of swabs and tissues, as previously described (17).

### Animals and procedures.

Mice were maintained in a specific-pathogen-free environment and were transferred to a containment facility after infection. Male and female C57BL/6 mice (8 to 15 weeks of age) were used for all experiments. Mice were bred in-house. Wild-type (WT) mice were acquired from Jackson Laboratory, and *Tnfrsf14^−/−^* mice (HVEM KO mice) were obtained from Yang-Xin Fu (39).

For ocular inoculation, mice were anesthetized with an intraperitoneal injection of ketamine/xylazine solution. Corneas were abraded lightly with a 25-gauge needle in a crosshatch pattern, and 5 μl of 2 × 10^6^ PFU HSV-1 strain 17 was applied to each cornea. Mice were weighed and scored for clinical symptoms daily. Clinical scores were divided into observations of lesions and of neurological symptoms. The scoring scale, which was also used in our previous studies, is shown in Table 1.

Mice displaying a neurological score of 4 or a loss of weight of greater or equal to 30% of the starting weight at infection were sacrificed and given a score of 5. Eye swabs were collected after mild anesthesia with isoflurane when mice were unresponsive to footpad prick. The eye was gently proptosed, and a sterile cotton swab premoistened with Dulbecco’s modified Eagle’s medium (DMEM) was wiped three times around the circumference of the eye and twice across the center of the cornea in an “X” shape as previously described (16). Swabs were then placed into 1 ml of DMER media (DMEM containing 5% [vol/vol] fetal bovine serum [FBS], 1% gentamicin, 1% ciprofloxacin, and 1% amphotericin B) and stored at −80°C. To determine viral titers, samples were thawed and subjected to vigorous vortex mixing for 30 s prior to performance of a plaque assay.

Two experimental groups of mice were used. In the first group, mice were sacrificed on day 5 for flow cytometry and eye swab procedures and for harvest of tissues. The mice in the second group were monitored for clinical symptoms, eye swabs were taken for viral titers, and mice were sacrificed for flow cytometry on day 14. For the day 5 experiments, animals were sacrificed and the POS, TG, and brain samples were collected as previously described (6, 7, 17). After dissection, all samples were placed in 1 ml DMER media, homogenized, sonicated, and stored at −80°C until titration. Brain samples were centrifuged prior to titration to remove debris.

### Flow cytometry.

On either day 5 or day 14 postinfection, corneal pairs and spleens were collected from individual mice and placed into cold phosphate-buffered saline (PBS). Corneas were placed into Liberase (Roche, Indianapolis, IN, USA) (0.7 mg/ml)–RPMI media for digestion for 1 h in an incubator (37°C, 5% CO_2_). Corneas were homogenized through a 100-μm-pore-size mesh with a 1-ml syringe plunger. Cells were washed with cold PBS, centrifuged at 4˚C, and then strained through a 40-μm-pore-size mesh and collected into 300-μl volumes. Spleens were prepared in a manner similar that used with the corneas but with an added red blood cell lysis incubation step performed between the 100-μm-pore-size-mesh and 40-μm-pore-size-mesh straining steps. Spleens were collected in 3-ml volumes. Counts of live cells were obtained using a Countess cell counter. All of the cornea cells and a portion of the spleen cells were each incubated in PBS at 1:1,000 using a Live/Dead Fixable Aqua dead cell stain kit (Thermo Fisher Scientific) in the dark at room temperature (RT) for 30 min. Samples were washed with PBS and incubated with Fc block (0.5 to 1.0 μg/sample anti-mouse CD16/CD32 [eBioscience, San Diego, CA, USA]–PBS–1% fetal bovine serum–0.1% sodium azide [fluorescence-activated cell sorter {FACS} buffer]) for 5 min at 48°C in the dark. Conjugated antibodies (2 μg/ml final concentration per sample) were added directly to Fc block and then incubated for 1 h at 4˚C in the dark.

The following antibodies were used: HVEM anti-mouse allophycocyanin (APC) (HMHV-1B18), Ly6G brilliant violet 421 (1A8), CD4 phycoerythrin (PE) (RM4-5), and CD8a brilliant violet 421 (53-6.7) from BioLegend; CD45 fluorescein isothiocyanate (FITC) (30-F11), CD3 APC-eFluor 780 (17A2), Cd11b PE Cy7 (M1/70), Cd11c PE (N418), Ly6C peridinin chlorophyll protein (PerCP) Cy5 (HK1.4), and CD3e PE Cy7 (145-2C11) from Ebioscience; and NK1.1 APC Cy7 (PK136) from BD Biosciences. The isotype controls (all from Ebioscience) were as follows: Armenian hamster (Arm Ham) IgG isotype control APC (eBio299Arm), rat IgG2b k isotype control PerCP eFluor 710 (eB149/10HS), and rat IgG2a isotype control APC eFluor 780 (eBR2a). Sample data were acquired using a FACSCanto II analyzer (BD Biosciences), the entire set of corneal pair samples was run, and the spleen sample run was stopped at 100,000 live cells. FlowJo 10.1 software (FlowJo, Ashland, OR, USA) was used for data analysis. All samples were first gated on lymphocytes. Single cells were separated according to the forward scatter corresponding to the area of the peak (FSC-A) versus the forward scatter corresponding to the height of the peak (FSC-H). Live cells were isolated using the Live/Dead plot. Cell populations were measured via flow cytometry gating on populations of CD45^+^ cells. CD45^+^/CD11b^+^ myeloid lineages were gated into further populations of Ly6G-/CD11c^+^ myeloid dendritic cells (mDCs), Ly6G^−^ CD11c^−^ monocytes and macrophages (M), Ly6G^−^/CD11c^−^/Ly6C^+^ inflammatory macrophages (I-M), Ly6G^−^/CD11c^−^/Ly6C^−^ noninflammatory macrophages (I-M), and Ly6G^+^/CD11c^+^ polymorphonuclear leukocytes (PMN). CD45^+^/CD3^+^ lymphoid lineages were gated into further populations of CD4^+^ CD8^−^ CD4 T cells (CD4^+^), CD4^−^ CD8^+^ CD8 T cells (CD8^+^), NK1.1^+^ natural killer T cells (NKT), and CD4^−^ CD8^−^ NK1.1^−^ double-negative T cells (DNT).

### Corneal sensitivity.

To determine the sensitivity of the central cornea, a Luneau Cochet-Bonnet esthesiometer (catalog no. WO-7760; Western Ophthalmics, Lynnwood, WA, USA) was used to measure the blink threshold. Animals were scruffed, and the length of the monofilament was adjusted from 6.0 to 0.5 cm and applied perpendicularly to the surface of the central cornea until the first inflection point. A positive response was recorded when two or more blinks were obtained in three attempts. Mice with an absence of a blink response at 0.5 cm were given a score of 0. The same examiner performed all measurements.

### Statistics.

Geometric means of numbers of viral PFU per tissue sample and maximum neurologic and lesion scores were compared using the unpaired Student's *t* test with the Holm-Sidak multiple-comparison test. The unpaired Student's *t* test and the Holm-Sidak multiple-comparison test were used to determine levels of variance between groups over time with respect to development of lesions or neurologic morbidity. GraphPad Prism 7.0 software was used for statistical analyses.

## References

[1] Geurs TL, Hill EB, Lippold DM, French AR 2012 Sex differences in murine susceptibility to systemic viral infections. J Autoimmun 38:J245–J253. doi:10.1016/j.jaut.2011.12.003. 22209097 PMC3313007

[2] Klein SL 2012 Sex influences immune responses to viruses, and efficacy of prophylaxis and treatments for viral diseases. Bioessays 34:1050–1059. doi:10.1002/bies.201200099. 23012250 PMC4120666

[3] Lundberg P, Welander P, Openshaw H, Nalbandian C, Edwards C, Moldawer L, Cantin E 2003 A locus on mouse chromosome 6 that determines resistance to herpes simplex virus also influences reactivation, while an unlinked locus augments resistance of female mice. J Virol 77:11661–11673. doi:10.1128/JVI.77.21.11661-11673.2003. 14557652 PMC229335

[4] Ngun TC, Ghahramani N, Sanchez FJ, Bocklandt S, Vilain E 2011 The genetics of sex differences in brain and behavior. Front Neuroendocrinol 32:227–246. doi:10.1016/j.yfrne.2010.10.001. 20951723 PMC3030621

[5] Wald A, Corey L 2007 Persistence in the population: epidemiology, transmission *In* Arvin A, Campadelli-Fiume G, Mocarski E, Moore PS, Roizman B, Whitley R, Yamanishi K (ed), Human herpesviruses: biology, therapy, and immunoprophylaxis. Cambridge University Press, Cambridge, United Kingdom. 21348071

[6] Fleming DT, McQuillan GM, Johnson RE, Nahmias AJ, Aral SO, Lee FK, St Louis ME 1997 Herpes simplex virus type 2 in the United States, 1976 to 1994. N Engl J Med 337:1105–1111. doi:10.1056/NEJM199710163371601. 9329932

[7] Edwards RG, Longnecker R 2017 Herpesvirus entry mediator and ocular herpesvirus infection: more than meets the eye. J Virol 91:e00115-17. doi:10.1128/JVI.00115-17. 28404853 PMC5469272

[8] Edwards RG, Kopp SJ, Ifergan I, Shui JW, Kronenberg M, Miller SD, Longnecker R 2017 Murine corneal inflammation and nerve damage after infection with HSV-1 are promoted by HVEM and ameliorated by immune-modifying nanoparticle therapy. Invest Ophthalmol Vis Sci 58:282–291. doi:10.1167/iovs.16-20668. 28114589 PMC5256684

[9] Liesegang TJ, Melton LJ III, Daly PJ, Ilstrup DM 1989 Epidemiology of ocular herpes simplex. Incidence in Rochester, Minn, 1950 through 1982. Arch Ophthalmol 107:1155–1159. doi:10.1001/archopht.1989.01070020221029. 2787981

[10] Clayton JA, Davis AF 2015 Sex/gender disparities and women’s eye health. Curr Eye Res 40:102–109. doi:10.3109/02713683.2014.986333. 25548854

[11] Thomas J, Gangappa S, Kanangat S, Rouse BT 1997 On the essential involvement of neutrophils in the immunopathologic disease: herpetic stromal keratitis. J Immunol 158:1383–1391. 9013983

[12] Torcia MG, Nencioni L, Clemente AM, Civitelli L, Celestino I, Limongi D, Fadigati G, Perissi E, Cozzolino F, Garaci E, Palamara AT 2012 Sex differences in the response to viral infections: TLR8 and TLR9 ligand stimulation induce higher IL10 production in males. PLoS One 7:e39853. doi:10.1371/journal.pone.0039853. 22768144 PMC3387221

[13] Rowe AM, St Leger AJ, Jeon S, Dhaliwal DK, Knickelbein JE, Hendricks RL 2013 Herpes keratitis. Prog Retin Eye Res 32:88–101. doi:10.1016/j.preteyeres.2012.08.002. 22944008 PMC3529813

[14] Norose K, Yano A, Zhang XM, Blankenhorn E, Heber-Katz E 2002 Mapping of genes involved in murine herpes simplex virus keratitis: identification of genes and their modifiers. J Virol 76:3502–3510. doi:10.1128/JVI.76.7.3502-3510.2002. 11884574 PMC136007

[15] Toma HS, Murina AT, Areaux RG Jr, Neumann DM, Bhattacharjee PS, Foster TP, Kaufman HE, Hill JM 2008 Ocular HSV-1 latency, reactivation and recurrent disease. Semin Ophthalmol 23:249–273. doi:10.1080/08820530802111085. 18584563

[16] Karaba AH, Kopp SJ, Longnecker R 2012 Herpesvirus entry mediator is a serotype specific determinant of pathogenesis in ocular herpes. Proc Natl Acad Sci U S A 109:20649–20654. doi:10.1073/pnas.1216967109. 23184983 PMC3528501

[17] Edwards RG, Kopp SJ, Karaba AH, Wilcox DR, Longnecker R 2015 Herpesvirus entry mediator on radiation-resistant cell lineages promotes ocular herpes simplex virus 1 pathogenesis in an entry-independent manner. mBio 6:e01532-15. doi:10.1128/mBio.01532-15. 26489863 PMC4620471

[18] Mo R, Chen J, Grolleau-Julius A, Murphy HS, Richardson BC, Yung RL 2005 Estrogen regulates CCR gene expression and function in T lymphocytes. J Immunol 174:6023–6029. doi:10.4049/jimmunol.174.10.6023. 15879095

[19] Kubarek L, Jagodzinski PP 2007 Epigenetic up-regulation of CXCR4 and CXCL12 expression by 17 beta-estradiol and tamoxifen is associated with formation of DNA methyltransferase 3B4 splice variant in Ishikawa endometrial adenocarcinoma cells. FEBS Lett 581:1441–1448. doi:10.1016/j.febslet.2007.02.070. 17362937

[20] Han X, Lundberg P, Tanamachi B, Openshaw H, Longmate J, Cantin E 2001 Gender influences herpes simplex virus type 1 infection in normal and gamma interferon-mutant mice. J Virol 75:3048–3052. doi:10.1128/JVI.75.6.3048-3052.2001. 11222734 PMC115935

[21] Shimeld C, Hill TJ, Blyth WA, Easty DL 1990 Reactivation of latent infection and induction of recurrent herpetic eye disease in mice. J Gen Virol 71:397–404. doi:10.1099/0022-1317-71-2-397. 2155293

[22] Zhang X, Castelli FA, Zhu X, Wu M, Maillere B, BenMohamed L 2008 Gender-dependent HLA-DR-restricted epitopes identified from herpes simplex virus type 1 glycoprotein D. Clin Vaccine Immunol 15:1436–1449. doi:10.1128/CVI.00123-08. 18667634 PMC2546687

[23] Klein SL, Flanagan KL 2016 Sex differences in immune responses. Nat Rev Immunol 16:626–638. doi:10.1038/nri.2016.90. 27546235

[24] Araneo BA, Dowell T, Diegel M, Daynes RA 1991 Dihydrotestosterone exerts a depressive influence on the production of interleukin-4 (IL-4), IL-5, and gamma-interferon, but not IL-2 by activated murine T cells. Blood 78:688–699. 1830499

[25] Barrat F, Lesourd B, Boulouis HJ, Thibault D, Vincent-Naulleau S, Gjata B, Louise A, Neway T, Pilet C 1997 Sex and parity modulate cytokine production during murine ageing. Clin Exp Immunol 109:562–568. doi:10.1046/j.1365-2249.1997.4851387.x. 9328137 PMC1904767

[26] Huber SA, Job LP, Auld KR 1982 Influence of sex hormones on coxsackie B-3 virus infection in Balb/c mice. Cell Immunol 67:173–179. doi:10.1016/0008-8749(82)90210-6. 6280880

[27] Barna M, Komatsu T, Bi Z, Reiss CS 1996 Sex differences in susceptibility to viral infection of the central nervous system. J Neuroimmunol 67:31–39. doi:10.1016/0165-5728(96)00022-7. 8707928

[28] Burgos JS, Ramirez C, Sastre I, Alfaro JM, Valdivieso F 2005 Herpes simplex virus type 1 infection via the bloodstream with apolipoprotein E dependence in the gonads is influenced by gender. J Virol 79:1605–1612. doi:10.1128/JVI.79.3.1605-1612.2005. 15650186 PMC544102

[29] Hill JM, Ball MJ, Neumann DM, Azcuy AM, Bhattacharjee PS, Bouhanik S, Clement C, Lukiw WJ, Foster TP, Kumar M, Kaufman HE, Thompson HW 2008 The high prevalence of herpes simplex virus type 1 DNA in human trigeminal ganglia is not a function of age or gender. J Virol 82:8230–8234. doi:10.1128/JVI.00686-08. 18550674 PMC2519549

[30] Labetoulle M, Auquier P, Conrad H, Crochard A, Daniloski M, Bouee S, El Hasnaoui A, Colin J 2005 Incidence of herpes simplex virus keratitis in France. Ophthalmology 112:888–895. doi:10.1016/j.ophtha.2004.11.052. 15878072

[31] Langenberg AG, Corey L, Ashley RL, Leong WP, Straus SE; Chiron HSV Vaccine Study Group. 1999 A prospective study of new infections with herpes simplex virus type 1 and type 2. N Engl J Med 341:1432–1438. doi:10.1056/NEJM199911043411904. 10547406

[32] Divito SJ, Hendricks RL 2008 Activated inflammatory infiltrate in HSV-1-infected corneas without herpes stromal keratitis. Invest Ophthalmol Vis Sci 49:1488–1495. doi:10.1167/iovs.07-1107. 18385067 PMC2367224

[33] Shui JW, Kronenberg M 2014 HVEM is a TNF receptor with multiple regulatory roles in the mucosal immune system. Immune Netw 14:67–72. doi:10.4110/in.2014.14.2.67. 24851095 PMC4022780

[34] Shui JW, Larange A, Kim G, Vela JL, Zahner S, Cheroutre H, Kronenberg M 2012 HVEM signalling at mucosal barriers provides host defence against pathogenic bacteria. Nature 488:222–225. doi:10.1038/nature11242. 22801499 PMC3477500

[35] Shui JW, Steinberg MW, Kronenberg M 2011 Regulation of inflammation, autoimmunity, and infection immunity by HVEM-BTLA signaling. J Leukoc Biol 89:517–523. doi:10.1189/jlb.0910528. 21106644 PMC3058819

[36] Ware CF, Sedy JR 2011 TNF superfamily networks: bidirectional and interference pathways of the herpesvirus entry mediator (TNFSF14). Curr Opin Immunol 23:627–631. doi:10.1016/j.coi.2011.08.008. 21920726 PMC3222271

[37] Azher TN, Yin XT, Tajfirouz D, Huang AJ, Stuart PM 2017 Herpes simplex keratitis: challenges in diagnosis and clinical management. Clin Ophthalmol 11:185–191. doi:10.2147/OPTH.S80475. 28176902 PMC5261835

[38] Darougar S, Wishart MS, Viswalingam ND 1985 Epidemiological and clinical features of primary herpes simplex virus ocular infection. Br J Ophthalmol 69:2–6. doi:10.1136/bjo.69.1.2. 3965025 PMC1040512

[39] Wang Y, Subudhi SK, Anders RA, Lo J, Sun Y, Blink S, Wang Y, Wang J, Liu X, Mink K, Degrandi D, Pfeffer K, Fu YX 2005 The role of herpesvirus entry mediator as a negative regulator of T cell-mediated responses. J Clin Invest 115:711–717. doi:10.1172/JCI200522982. 15696194 PMC546456

