# Erratum for Riccio et al., “Characterization of Sex Differences in Ocular Herpes Simplex Virus 1 Infection and Herpes Stromal Keratitis Pathogenesis of Wild-Type and Herpesvirus Entry Mediator Knockout Mice”

**DOI:** 10.1128/mSphere.00322-19

**Published:** 2019-05-15

**Authors:** Rachel E. Riccio, Seo J. Park, Richard Longnecker, Sarah J. Kopp

**Affiliations:** a Department of Microbiology and Immunology, Northwestern University Feinberg School of Medicine, Chicago, Illinois, USA

## ERRATUM

Volume 4, issue 2, e00073-19, 2019, https://doi.org/10.1128/mSphere.00073-19. In the 2nd sentence of the importance section (PDF page 1, second paragraph) and the 1st sentence of the 2nd paragraph of the main text (PDF page 2, first full paragraph), herpesvirus stromal keratitis should be described as “a leading cause of infectious blindness worldwide” rather than “the leading cause of infectious blindness worldwide.”

